# Neutrophil Gelatinase-Associated Lipocalin Increases HLA-G^+^/FoxP3^+^ T-Regulatory Cell Population in an In Vitro Model of PBMC

**DOI:** 10.1371/journal.pone.0089497

**Published:** 2014-02-27

**Authors:** Gaetano La Manna, Giulia Ghinatti, Pier Luigi Tazzari, Francesco Alviano, Francesca Ricci, Irene Capelli, Vania Cuna, Paola Todeschini, Eugenio Brunocilla, Pasqualepaolo Pagliaro, Laura Bonsi, Sergio Stefoni

**Affiliations:** 1 Dialysis, Nephrology and Transplantation Unit, Department of Experimental, Diagnostic and Specialty Medicine, St. Orsola University Hospital, Bologna, Italy; 2 Department of Internal and Experimental Medicine, Second University of Studies of Naples, Naples, Italy; 3 Service of Immunohematology and Transfusion Medicine, Department of Hematology, Oncology and Laboratory Medicine, St. Orsola University Hospital, Bologna, Italy; 4 Section of Histology, Embryology and Applied Biology, Department of Experimental, Diagnostic and Specialty Medicine, University of Bologna, Bologna, Italy; 5 Urology Unit, Department of Experimental, Diagnostic and Specialty Medicine, St. Orsola University Hospital, Bologna, Italy; Tulane University, United States of America

## Abstract

**Background:**

Neutrophil gelatinase-associated lipocalin (NGAL) is emerging as a mediator of various biological and pathological states. However, the specific biological role of this molecule remains unclear, as it serves as a biomarker for many conditions. The high sensitivity of NGAL as a biomarker coupled with relatively low specificity may hide important biological roles. Data point toward an acute compensatory, protective role for NGAL in response to adverse cellular stresses, including inflammatory and oxidative stress. The aim of this study was to understand whether NGAL modulates the T-cell response through regulation of the human leukocyte antigen G (HLA-G) complex, which is a mediator of tolerance.

**Methodology/Principal Findings:**

Peripheral blood mononuclear cells (PBMCs) were obtained from eight healthy donors and isolated by centrifugation on a Ficoll gradient. All donors gave informed consent. PBMCs were treated with four different concentrations of NGAL (40–320 ng/ml) in an iron-loaded or iron-free form. Changes in cell phenotype were analyzed by flow cytometry. NGAL stimulated expression of HLA-G on CD4+ T cells in a dose- and iron-dependent manner. Iron deficiency prevented NGAL-mediated effects, such that HLA-G expression was unaltered. Furthermore, NGAL treatment affected stimulation of regulatory T cells and in vitro expansion of CD4^+^ CD25^+^ FoxP3^+^ cells. An NGAL neutralizing antibody limited HLA-G expression and significantly decreased the percentage of CD4^+^ CD25^+^ FoxP3^+^ cells.

**Conclusions/Significance:**

We provide *in vitro* evidence that NGAL is involved in cellular immunity. The potential role of NGAL as an immunomodulatory molecule is based on its ability to induce immune tolerance by upregulating HLA-G expression and expansion of T-regulatory cells in healthy donors. Future studies should further evaluate the role of NGAL in immunology and immunomodulation and its possible relationship to immunosuppressive therapy efficacy, tolerance induction in transplant patients, and other immunological disorders.

## Introduction

Neutrophil gelatinase-associated lipocalin (NGAL) is a glycoprotein belonging to the family of “lipocalins,” which are small secreted molecules that maintain health and prevent diseases. NGAL has recently been reported to be a biomarker of various benign and malignant conditions and has emerged as an attractive molecular tool with distinct clinical applications for diagnosis and follow up of several diseases [1].

The functions of NGAL in pathological processes include modulation of intracellular stores of iron [2], bacteriostatic activity [3], and a potential role in inflammation [1]. In particular, evidence has emerged suggesting NGAL effects in (i) neutrophil chemotaxis [3] and (ii) antagonizing oxidative stress [4]. The assay for this molecule shows extreme sensitivity but is associated with low specificity [5]. NGAL has become a successful biomarker for functional, toxic, and ischemic renal damage [6] and for cardiorenal syndromes [7], [8]. Concentrations of this small peptide increase in several conditions. Soluble NGAL has been shown to increase with bacterial infections, inflammatory and ischemic damage, metabolic disease, kidney disease, drug and pathogen intoxications, and solid and hematological malignancies [9], [10]. Significantly elevated NGAL has also been described in embryo conditions [11], stem cells [12], and as a result of organ transplants [13].

The biological role of this molecule is unclear. Although NGAL serves as a biomarker for many conditions, it is evident that high sensitivity is associated with low specificity.

The potential immunological effects are not thoroughly understood, but there is evidence that NGAL may be associated with inflammatory mechanisms [1].

The potential role of NGAL in inflammatory processes, including modulation of the immune response, should be investigated. An inflammatory role may activate processes that counteract aggressive conditions, such as bacterial infection, ischemic damage, apoptosis, and necrosis. Furthermore, NGAL may mediate active anti-inflammatory processes that promote regeneration, repair, and restoration of stable conditions.

Recently the interaction between NGAL and NF-κb and its involvement in the innate and adaptive immune systems has been studied, suggesting a possible role for NGAL in immune tolerance [14], [15].

HLA-G is a non-classical HLA class I molecule with an important role at the fetal-maternal interface, preventing fetus recognition and abortion. The genetic diversity, expression, structure, and function of HLA-G differs from HLA I molecules: it does not appear to significantly stimulate the immune system. However, like HLA class I molecules, HLA-G is able to bind to inhibitory receptors. It is currently considered a key molecule in the complex still not entirely understood phenomenon of tolerance [16].

The aims of the present study were to evaluate the potential immunomodulatory role of NGAL. In particular, we tested the effect of NGAL in an *in vitro* model of peripheral blood mononuclear cells (PBMCs). We evaluated the expression of human leukocyte antigen G (HLA-G), a well-known tolerogenic molecule, and the presence of a FoxP3+ T-regulatory cell subset.

## Materials and Methods

### Ethics Statement

For the following analysis we recruited 8 healthy volunteers. The study was approved by the local ethics committee (S.Orsola-Malpighi University Hospital, Bologna, Italy protocol number: 33/2013/U/Sper) and written informed consent was obtained from all subjects.

### Flow cytometry analysis of PBMC

Flow cytometry analysis of CD4 and CD8 on the surfaces of PBMCs was performed with PC5-anti-CD4 monoclonal antibody (mAb) (Beckman-Coulter, Brea CA) and PE-anti-CD8 mAb (Beckman-Coulter, Brea CA). These antibodies were diluted 1/20 in PBS and added to 10^5^ cells for 20 minutes at room temperature. HLA-G expression on CD4 and CD8 mAb-labeled PBMC was detected after cell fixation and permeabilization with the Intraprep Kit (Beckman-Coulter) and by staining with the mAb MEMG/9-FITC (Abcam, Cambridge, UK) for 20 minutes at room temperature.

Detection of CD4+ CD25+ FoxP3+ regulatory T cells on PBMC was performed with the Treg Detection Kit, CD4/CD25/FoxP3 (Miltenyi, Bergish Gladbach, Germany) as described in the manufacturer's protocol. Briefly, after fixation, PBMCs were incubated for 10 minutes in the dark in the refrigerator (2–8°C) with CD4-FITC mAb and CD25-APC mAb. After permeabilization, cells were incubated for 30 minutes in the dark in the refrigerator (2–8°C) with an anti-FoxP3-PE antibody (Miltenyi, Bergish Gladbach, Germany).

Co-expression of CD4, HLA-G, and FoxP3 was examined in previously fixed cells that were labeled with anti-CD4-PC5, permeabilized with the Intraprep Kit (Beckman-Coulter), and stained with MEMG/9-FITC mAb and FoxP3-PE mAb.

Negative controls were stained with appropriately conjugated irrelevant antibodies. Cells were extensively washed in PBS and analyzed with a Navios Flow Cytometer (Beckman-Coulter). Ten thousand events were acquired. Results were analyzed with Kaluza Software (Beckman-Coulter).

### PBMC isolation and culture

PBMCs were obtained from eight healthy donors. Blood samples of 30 ml were obtained and placed in EDTA-anticoagulant tubes. PBMCs were isolated from whole blood by Ficoll gradient (GE Healthcare Bio-Sciences AB, Uppsala, Sweden) and resuspended in RPMI media (Lonza, Basel, Switzerland) with 10% fetal bovine serum (Lonza), 100 U/mL penicillin, and 100 U/mL streptomycin (Sigma-Aldrich, St Louis, MO, USA). PBMCs were activated with 5 µg/ml phytohemagglutinin (PHA; Sigma Aldrich) in cell culture.

### NGAL treatment of PBMCs and HLA-G expression

PHA-activated PBMCs were treated with NGAL (R&D Systems, Minneapolis, USA), conjugated to Enterobactin:Iron complex (ECM Microcollection, Tuebingen, Germany). Cells were treated with increasing concentrations of NGAL:Enterobactin:Iron (40 ng/ml, 80 ng/ml, 160 ng/ml, and 320 ng/ml) and incubated at 37°C and 5% CO_2_ for 72 hours. In order to evaluate if HLA-G expression was directly modulated by NGAL, cells were incubated with 40 ng/ml NGAL conjugated to iron-free enterobactin and with 40 ng/ml Enterobactin:Iron complex at 37°C and 5% CO_2_ for 72 hours. Expression of HLA-G was evaluated as specified above.

We also evaluated if HLA-G expression depends on an NGAL effect. NGAL activity was inhibited with an anti-NGAL mAb (200 µg; 1 mg/ml) (Thermo Scientific, Waltham, MA, USA). Cells were treated with 25 µl/ml anti-NGAL (50 ng/ml) and 50 µl/ml anti-NGAL (100 ng/ml) to inhibit 40 ng/ml NGAL and 80 ng/ml NGAL, respectively.

### NGAL treatment of PBMCs and CD4^+^ CD25^+^ FoxP3^+^ cell expression

PHA-activated PBMCs were treated with increasing concentrations of NGAL:Enterobactin:Iron (40 ng/ml, 80 ng/ml, 160 ng/ml, and 320 ng/ml). Cells were incubated at 37°C and 5% CO_2_ for 72 hours.

In order to evaluate if CD4+ CD25+ FoxP3+ cell expression was directly modulated by NGAL, cells were incubated with 40 ng/ml NGAL conjugated to iron-free enterobactin and with 40 ng/ml Enterobactin:Iron complex at 37°C and 5% CO_2_ for 72 hours. The percentage of CD4^+^ CD25^+^ FoxP3^+^ cells was determined by the Treg Detection Kit (CD4/CD25/FoxP3-PE; Miltenyi Biotech).

We also evaluated if the CD4^+^ CD25^+^ FoxP3^+^ cell percentage depends on NGAL activity. NGAL activity was inhibited with an anti-NGAL mAb (200 µg; 1 mg/ml) (Thermo Scientific, Waltham, MA, USA). Cells were treated with 25 µl/ml anti-NGAL (50 ng/ml) and 50 µl/ml anti-NGAL (100 ng/ml) to inhibit 40 ng/ml NGAL and 80 ng/ml NGAL, respectively.

### sHLA-G production by PBMCs after NGAL treatment

PBMCs stimulated with PHA were cultured with 40–320 ng/ml NGAL:Enterobactin:Iron and 40 ng/ml NGAL:Enterobactin. After 72 h of incubation the culture supernatants were collected and analyzed for detection of sHLA-G with ELISA (BioVendor, Prague, Czech Republic).

### Statistical Analysis

The results are presented as mean (from the number of samples indicated) ± SD. Two-tailed t tests were conducted to determine statistical significance.

## Results

### HLA-G expression in PHA-activated PBMCs treated with NGAL:enterobactin:iron complex

**Figure 1:**
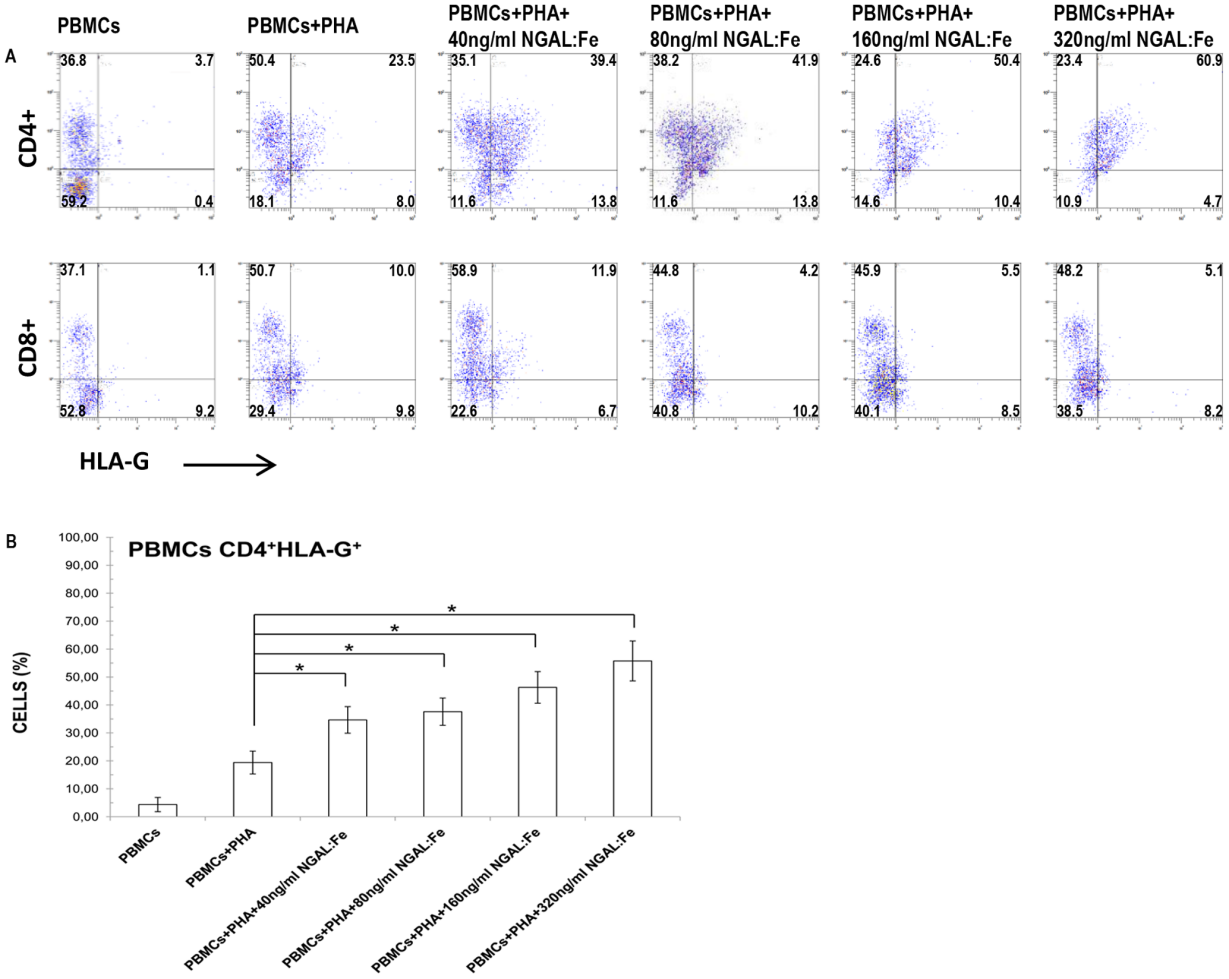
Effect of NGAL on HLA-G expression in PHA-activated PBMCs. A) Flow cytometry analysis of surface HLA-G on CD4^+^ and CD8^+^ cells. PBMCs were treated with increasing concentrations of NGAL:Enterobactin:Iron (40 ng/ml, 80 ng/ml, 160 ng/ml, and 320 ng/ml). A dose-dependent rise in the percentage of CD4^+^HLA-G^+^ cells was evident. The percentage of CD8^+^HLA-G^+^ cells did not show any marked changes. Data are representative of 8 independent experiments. The number in each quadrant represents the percentage of the total population. B) Data are expressed as percentages of CD4^+^ HLA-G^+^ cells. Means ± SD; n = 8. * p<0.05.

We examined the NGAL effect on HLA-G expression in CD4^+^ and CD8^+^ cell populations. PBMCs were treated with increasing concentrations of NGAL:Enterobactin:Iron (40 ng/ml, 80 ng/ml, 160 ng/ml, and 320 ng/ml) and HLA-G expression was evaluated on CD4^+^ and CD8^+^ cells. We observed a dose-dependent increase in HLA-G expression on CD4^+^ (range of means, 34.7±5.8% – 55.8+7.2%; n = 8) as compared to unactivated PBMCs (mean, 3.8±0.2%) and PHA-activated PBMCs (mean, 19.4±4.1%). On the contrary, CD8^+^ cells did not show any increase in HLA-G expression after treatment with increasing concentrations of NGAL:Enterobactin:Iron (range, 4.2%-11.9%; n = 8). These results indicated a different effect by NGAL on HLA-G expression in the cases of activated PBMC CD4^+^ and CD8^+^ cells. In addition, the rise in CD4^+^HLA-G^+^ cells was in proportion to the NGAL:Enterobactin:Iron concentration (40–320 ng/ml) (Figure 1A–B).

Concerning the levels of soluble-form HLA-G, ELISA results showed that following treatment with NGAL:Enterobactin:Iron complex, sHLA-G values increased in a dose-related manner up to a concentration of 80 ng/ml NGAL:Enterobactin:Iron. In samples treated with 160 ng/ml and 320 ng/ml NGAL:Enterobactin:Iron, levels of sHLA-G were reduced (respectively mean, 11.1±1.4 U/ml and 10.5±1.1 U/ml; n = 3) in comparison to 40 and 80 ng/ml NGAL:Enterobactin:Iron (respectively mean, 16.2±1.4 U/ml and 19.3±5.4 U/ml; n = 3). Stimulation with 40 ng/ml NGAL:Enterobactin complex (without iron) induced a higher level of sHLA-G (mean, 15.7+2.2 U/ml; n = 3) than occurred with controls of unactivated PBMCs and PHA-activated PBMCs (respectively mean, 3.8±2.6 U/ml and 6.9±1.2 U/ml; n = 3) (Figure S1).

### Evaluation of iron effect and NGAL neutralization on HLA-G expression in PHA-activated CD4^+^PBMCs

PBMCs were activated with PHA and treated with iron (without NGAL) and 40 ng/ml NGAL:enterobactin complex (without iron). Iron treatment alone did not increase the percentage of CD^+^HLA-G^+^ cells (mean, 19.7±3.6%; n = 8) in comparison to controls of PHA-activated PBMCs (mean, 19.5±4.1%; n = 8).

**Figure 2:**
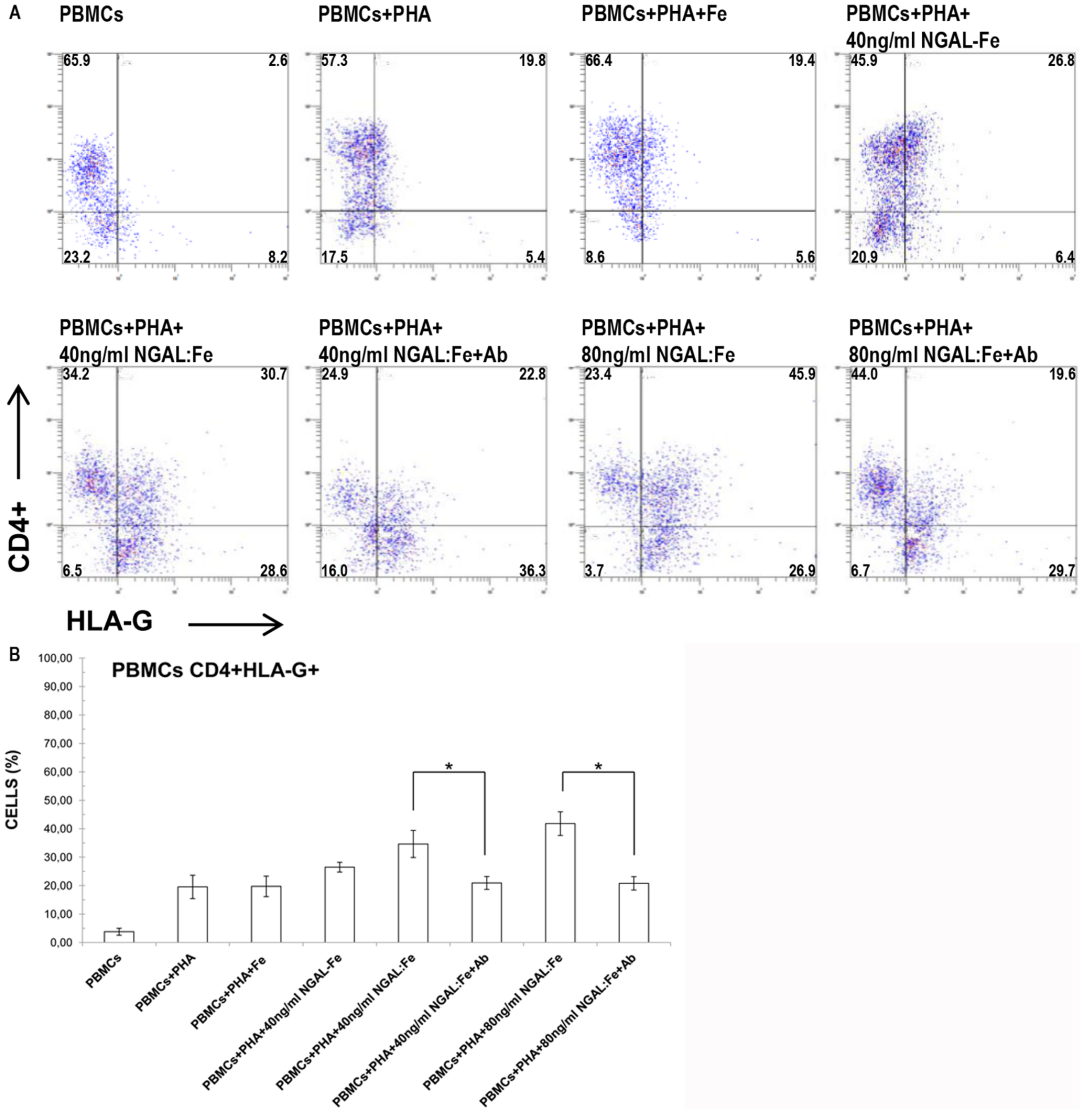
Effects of iron and NGAL neutralization on HLA-G expression in PHA-activated PBMCs. Flow cytometry analysis of HLA-G expression on CD4^+^ cells. Iron treatment (without NGAL) did not increase the percentage of HLA-G^+^ cells. Treatment with the NGAL:enterobactin complex (without iron) reduced HLA-G expression in contrast to stimulation with NGAL:Enterobactin:Iron. Incubation with anti-NGAL antibody reduced the percentage of HLAG^+^ cells. Data are representative of 8 independent experiments. The number in each quadrant represents the percentage of the total population. B) Data are expressed as percentages of CD4^+^ HLA-G^+^ cells. Means ± SD; n = 8. * p<0.05.

Treatment with NGAL:enterobactin complex (without iron) slightly raised the expression of CD4+HLA-G+ cells despite the absence of iron (mean, 26.5±1.7%; n = 8). These data suggest that the need for iron in order that there be HLA-G expression is unclear (Figure 2A–B).

Next, we treated PBMCs with an anti-NGAL mAb to inhibit the effects of NGAL stimulation. We found that NGAL-neutralizing antibody reduced the percentages of CD4+HLA-G+ cells. Respectively, the percentage of CD4+HLA-G+ cells, after treatment with 40 and 80 ng/ml NGAL:Enterobactin:Iron (mean, 34.7±4.8% and 41.8±4.2%; n = 8), significantly decreased when anti-NGAL mAb, at increasing doses (50 ng/ml and 100 ng/ml), was added to culture (mean, 20.9±2.3% and 20.8±3.4%; n = 8) (Figure 2A–B). These results confirmed that NGAL affects HLA-G expression in PHA-activated PBMC CD4^+^ cells.

### CD4^+^/CD25^+^/FoxP3^+^ cells in PHA-activated PBMCs treated with NGAL:enterobactin:iron complex

**Figure 3:**
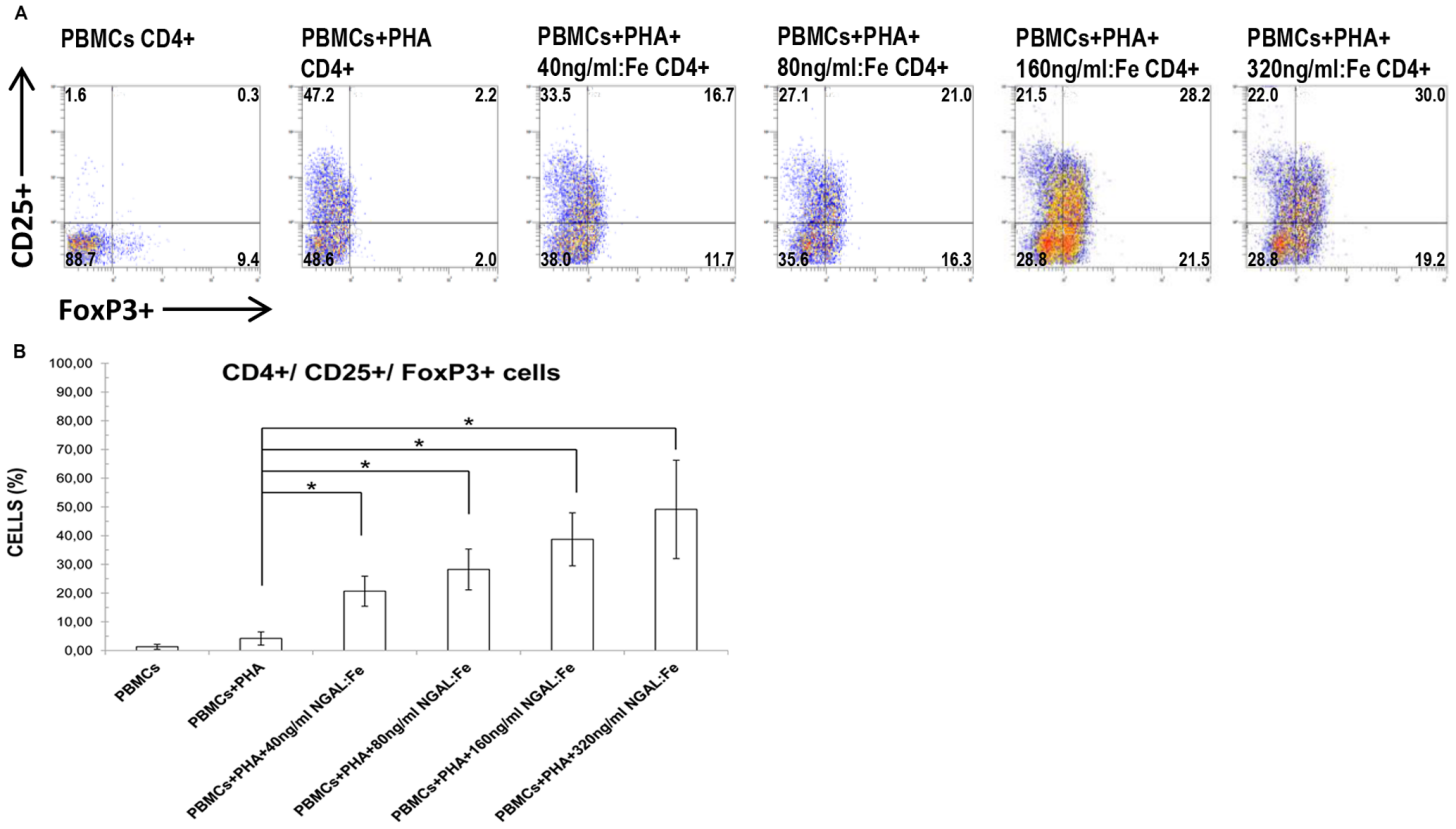
Effect of NGAL on the percentage of CD4^+^/CD25^+^/FoxP3^+^ cell population. **A).** The percentage of CD4^+^/CD25^+^/FoxP3^+^ cells in PHA-activated PBMCs after stimulation with increasing concentrations of NGAL: Enterobactin:Iron (40 ng/ml, 80 ng/ml, 160 ng/ml, and 320 ng/ml), raised in a dose dependent manner. Data are representative of 8 independent experiments. The number in each quadrant represents the percentage of CD25^+^/FoxP3^+^ cells on gated CD4^+^ cells of the total population. B) Data are expressed as percentages of CD4^+^/CD25^+^/FoxP3^+^ cells. Means ± SD; n  =  8. *p<0.05.

The role of NGAL in immune modulation appears also to involve regulatory T cell populations, which are identified by CD4^+^/CD25^+^/FoxP3^+^ expression. This is particularly interesting, because regulatory T cells have a pivotal role in inducing immune tolerance. We evaluated the percentages of CD4^+^/CD25^+^/FoxP3^+^ cells following NGAL:Enterobactin:Iron complex stimulation (40 ng/ml, 80 ng/ml, 160 ng/ml, and 320 ng/ml). We found a dose-dependent increase in the percentage of CD4^+^/CD25^+^/FoxP3^+^ cells (range of means, 20.7±5.2%–49.1+17.1%; n = 8) compared to unactivated PBMCs (mean, 1.3±0.9%) and PHA-activated PBMCs (mean, 4.2±2.3%) (Figure 3A–B).

### Evaluation of iron effect and NGAL neutralization on the CD4^+^/CD25^+^/FoxP3^+^ cell percentage in PHA-activated PBMCs

PBMCs were activated with PHA and treated with iron (without NGAL) and 40 ng/ml NGAL:enterobactin complex (without iron). Iron treatment alone did not increase the percentage of CD4^+^/CD25^+^/FoxP3^+^ cells (mean, 4.3±1.3%; n = 8) in comparison to the control of PHA-activated PBMCs (mean, 3.8±2.8%; n = 8).

**Figure 4:**
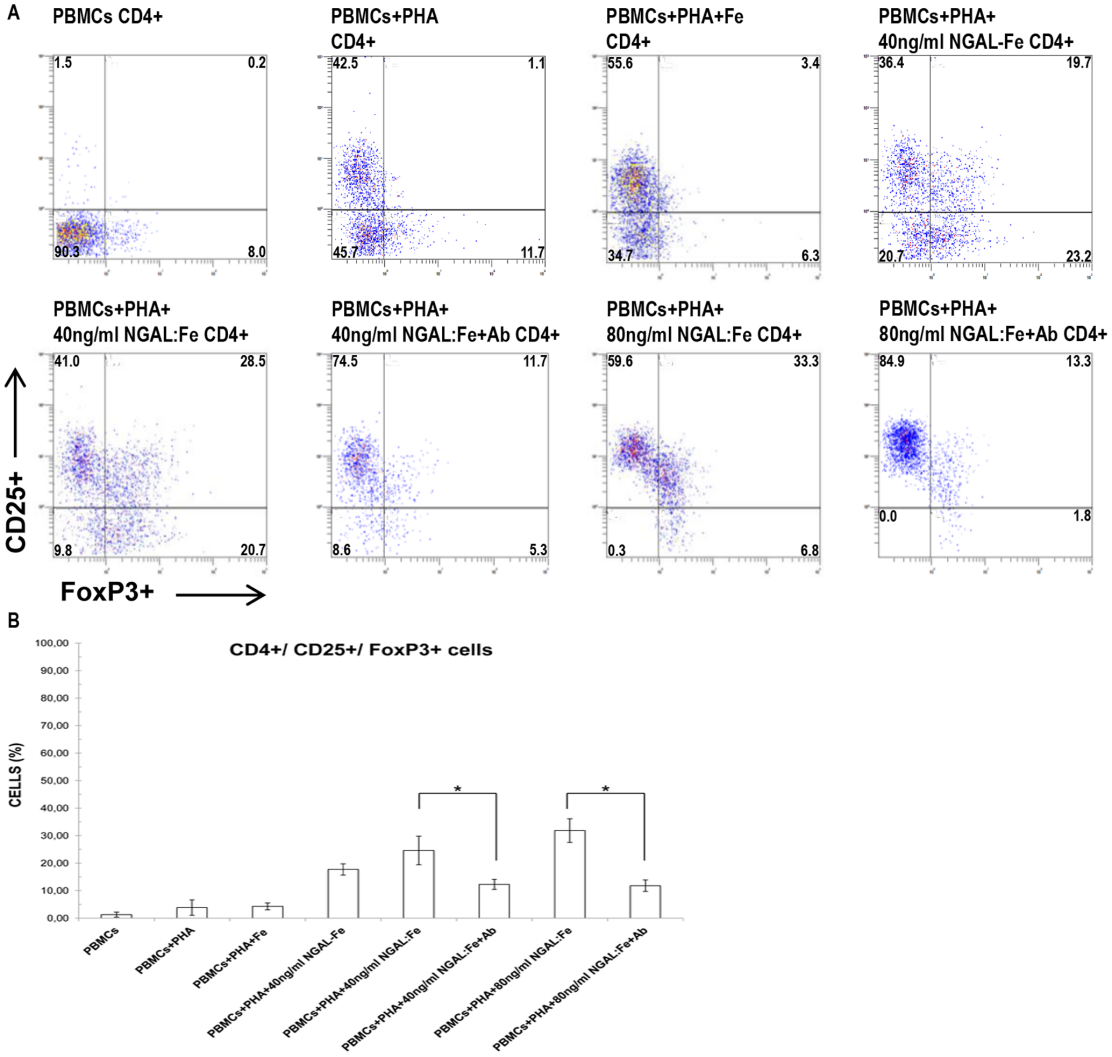
Effects of iron and NGAL neutralization on the percentage of CD4^+^/CD25^+^/FoxP3^+^ cell population. Flow cytometry analysis of CD25^+^/FoxP3^+^ expression on CD4^+^ cells in PHA-activated PBMCs. Iron treatment (without NGAL) did not increase the percentage of CD25^+^/FoxP3^+^ cells. Treatment with NGAL:enterobactin complex (without iron) reduced the percentage of CD25^+^/FoxP3^+^ cells in contrast to stimulation with NGAL:Enterobactin:Iron. Incubation with anti-NGAL antibody reduced the percentage of CD25^+^/FoxP3^+^ cells. Data are representative of 8 independent experiments. The number in each quadrant represents the percentage of CD25+foxP3 cells on gated CD4**^+^** cells of the total population. B) Data are expressed as percentages of CD4+/CD25+/FoxP3+ cells. Means ± SD; n = 8. * p<0.05.

By contrast, treatment with NGAL:enterobactin complex (without iron) raised the expression of CD4^+^/CD25^+^/FoxP3^+^ cells (mean, 17.7±2.1%; n = 8). These results showed that enterobactin and iron, not conjugated with NGAL, had no effect on the percentages of CD4^+^/CD25^+^/FoxP3^+^ cells (Figure 4A–B).

Cells were also treated with anti-NGAL mAb in order to confirm that NGAL stimulation was involved in the increased levels of CD4^+^/CD25^+^/FoxP3^+^ T-regulatory cells.

We found that the NGAL neutralizing antibody significantly reduced the percentages of CD4^+^/CD25^+^/FoxP3^+^ cells. Respectively, the percentage of CD4^+^/CD25^+^/FoxP3^+^ cells, after treatment with 40 and 80 ng/ml NGAL:Enterobactin:Iron (mean, 24.6±5.2% and 31.8±4.3%; n = 8), significantly decreased when increasing doses of anti-NGAL mAb (50 ng/ml and 100 ng/ml) were added to the culture (mean, 12.3±1.8% and 11.8±1.5%; n = 8) (Figure 4A–B). These results confirmed that NGAL affects the CD4^+^/CD25^+^/FoxP3^+^ cell percentage in PHA-activated PBMC cells.

### HLA-G^+^/FoxP3^+^ cells in PHA-activated PBMCs treated with NGAL:enterobactin:iron complex

**Figure 5:**
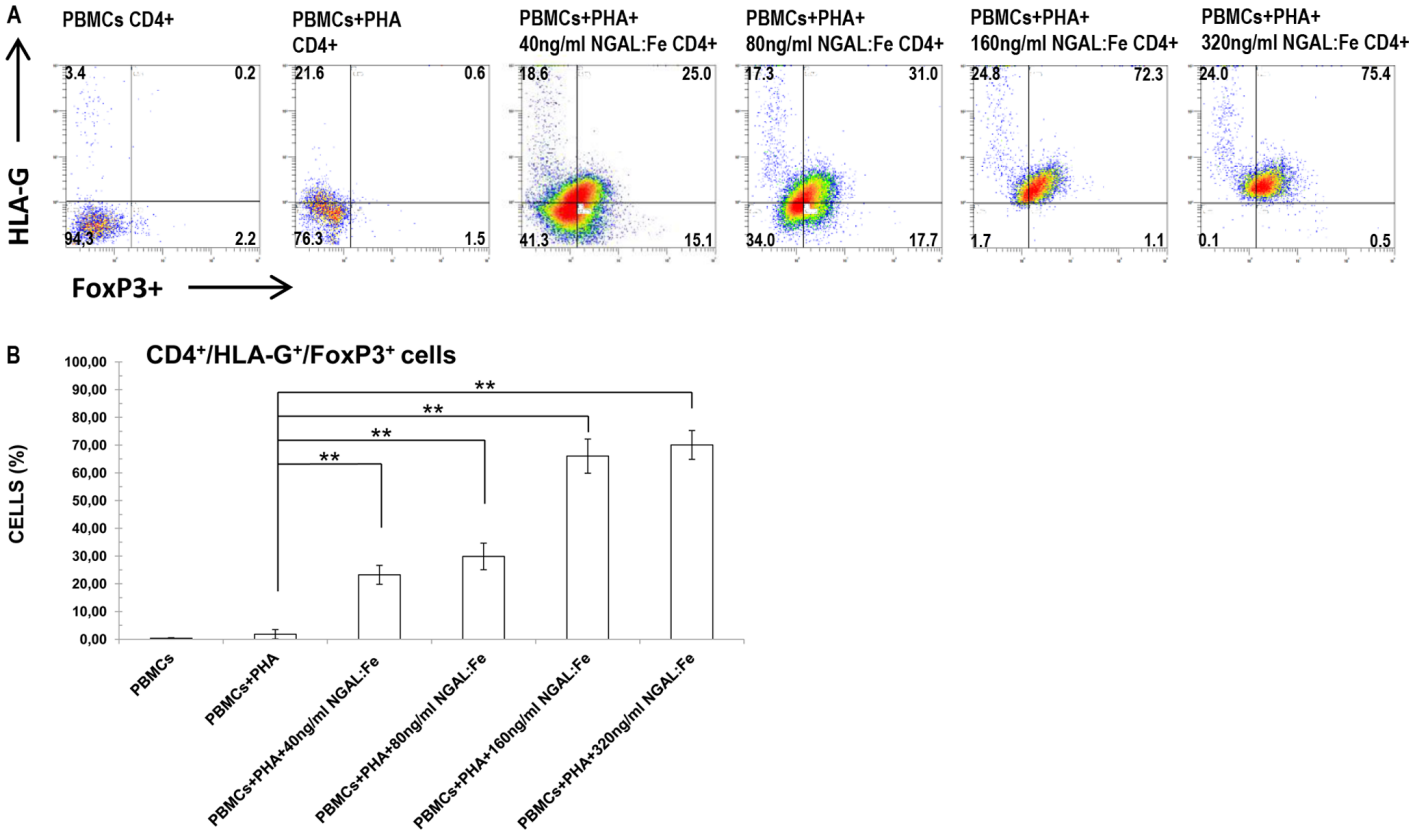
Effect of NGAL on the percentage of CD4^+^/HLA-G^+^/FoxP3^+^ cell population. Flow cytometry analysis of HLA-G**^+^**/FoxP3**^+^** expression on CD4^+^ cell PHA-activated PBMCs. PBMCs were treated with increasing concentrations of NGAL:Enterobactin:Iron (40 ng/ml, 80 ng/ml, 160 ng/ml, and 320 ng/ml). A dose-dependent raise in the percentage of HLA-G**^+^**/FoxP3**^+^** cells was evident. Data are representative of 8 independent experiments. The number in each quadrant represents the percentage of HLA-G**^+^**/FoxP3**^+^** cells on gated CD4**^+^** cells of the total population. B) Data are expressed as percentages of CD4**^+^**/HLA-G**^+^**/FoxP3**^+^** cells. Means ± SD; n = 8. * p<0.05.

In order to evaluate the effect of NGAL on HLA-G expression in CD4+/FoxP3+ cells following treatment with NGAL, we added scalar doses of NGAL:Enterobactin:Iron complex (40–320 ng/ml) to our cell cultures. The results showed that CD4**^+^**/HLA-G**^+^**/FoxP3**^+^** levels significantly increased in a dose-dependent manner in response to NGAL stimulation (Fig. 5A–B). We observed a dose-dependent rise in the CD4**^+^**/HLA-G**^+^**/FoxP3**^+^** cell percentage (range of means, 23.2.7±3.4%–70.1±5.2%; n = 8) compared to unactivated PBMCs (mean, 0.3±0.2%) and PHA-activated PBMCs (mean, 1.8±1.7%).

## Discussion/Conclusion

We have shown that NGAL increases the HLA-G expression in a dose-dependent manner. This result is consistent with reports in the literature, which suggest that NGAL protects against various different types of damage, ischemic, infectious and vascular. These functions are associated with antioxidant activity, which may require iron chelation. However, our data did not show a clear requirement for iron, although HLA-G expression in the absence of iron was slightly lower than in the presence of iron. Thus, the role of iron in NGAL activity should be further investigated. Other actions of NGAL, such as its function in the non-transferrin-bound serum iron (NTBI) family, cannot be excluded. For example, tubular cytoresistance has been described in association with antioxidant activity. NGAL is a major iron-transporting protein complementary to transferrin, and is one of the most highly-induced genes and proteins in the kidney following ischemic injury [17], [18]. Iron chelation helps reduce the amount of iron available, favouring its powerful antioxidant action. There is evidence for the role of reactive oxygen species (ROS) in the pathogenesis of acute kidney injury (AKI). ROS cause injury to renal tubule cells by protein oxidation, lipid peroxidation, DNA damage, and induction of apoptosis. In animal models several ROS scavengers protect against ischemic AKI. However, human studies have here been inconclusive [19]. Free iron derived from red cells or other injured cells is one of the most potent factors involved in ROS generation. For example, the iron scavenger deferoxamine alleviates ischemia-reperfusion injury in animal models [19].

Clinically employed iron formulations, such as iron sucrose (FeS), iron gluconate (FeG), and ferumoxytol, influence renal redox-sensitive signaling, cytotoxicity, and responses to superimposed stress. Parental iron formulations can induce cytotoxicity and nephrotoxicity. Secondary adaptive responses to this injury can induce renal tubular cytoresistance, suggesting a potential clinical application for iron therapy[20].

New advances have emerged in the field of iron chelation. NGAL belongs to the NTBI transporter family [21] which promotes differentiation and structural organization of renal epithelial cells [22]. Furthermore, NGAL is an early and quantitative urinary biomarker for cisplatin nephrotoxicity in mouse models [18].

NGAL provides protective effects in experimental models of AKI. Administration of NGAL in animal models before, during, or even shortly after ischemic injury contributes to the protection of tubule epithelial cells at the functional and structural levels with induction of proliferation and inhibition of apoptosis [18], [23]. Hence, during recovery from ischemic damage, renal tubular cells recapitulate processes that are very similar to those that occur during normal kidney development [24]–[26]. Importantly, NGAL is highly expressed in proliferating tubule cells, suggesting a protective or regenerative role after AKI. NGAL plays a critical role in kidney development during the conversion of kidney progenitors into epithelia and tubules, providing evidence for this notion [27]. NGAL provides a reservoir for excess iron and may supply a regulated source of intracellular iron to stimulate repair and regeneration. In this context, NGAL reduces iron-mediated toxicity.

Our data suggest that NGAL may activate an iron-independent pathway through a different receptor. Thus, NGAL may exert a parallel action to that resulting from simple iron chelation and intracellular iron transport. The NGAL effect on HLA-G expression could be direct but we can not exclude the possibility that it may be indirect and mediated by another intermediate factor.

Our data showed a dubious but possible action by iron in the expression of membrane-bound and soluble isoforms of HLA-G. We also observed that iron had no effect on FoxP3+ cell differentiation. Different mechanisms of cellular activation and up-regulation of FoxP3+ cells have been reported in the literature.

Exogenous administration of NGAL markedly up-regulates heme oxygenase-1, a multifunctional protective agent in experimental AKI. Heme oxygenase-1 reduces iron uptake, promoting intracellular iron release, stimulating production of antioxidants, and inducing expression of the cell cycle regulatory protein p21 [28].

The interaction between NGAL and nuclear factor-kappa B (NF-kB) has recently been studied. Expression of NGAL mRNA and protein is upregulated in an NF-kB-dependent manner in rat and human vascular smooth muscle cells in response to IL-1β stimulation [14]. The possible interaction between NF-kB and the NGAL gene requires IκB-ζ in order for it to be induced. Coexpression of IκB-ζ and NF-κB subunits synergistically activates transcription of NGAL genes [15].

The possible involvement of NGAL in the inflammatory process of human mesangial cells has been explored. *In vitro* experiments showed that NGAL receptor mRNA and protein levels were dramatically induced by treatment with IL-1β through activation of the MAPK/ERK pathway [29]. The transcription factor NF-κB plays a key role in the innate and adaptive immune systems. The interaction between NGAL and NF-Kb and the involvement of NF-κB in the innate and adaptive immune systems suggest a possible role for NGAL in immune tolerance.

Regulatory T cells have gained much interest within the research community and are considered ideal candidates for monitoring immune tolerance. Treg cells constitute 1–10% of thymic and peripheral CD4+ T cells in humans and mice, and arise during athymic selection. They are characterized by constitutive expression of the IL-2Rα chain (CD25) and expression of the forkhead winged helix transcriptional regulator Foxp3. The importance of Foxp3 has been demonstrated by natural mutations of the Foxp3 gene that result in a loss of Treg cell function and the development of severe autoimmune diseases [30]. The Treg cell population can be divided into the naturally occurring Foxp3 Treg population, generated in the thymus, and any of the many inducible Treg cell populations that are derived in the periphery from CD4+Foxp3− precursors upon activation in the presence of differentiating signals [31]. HLA-G–related regulatory cells exist and some of these can have a long-lasting inhibitory effect on immune responses. HLA-G–induced regulatory T cells were first observed after stimulation of T cells with HLA-G1–expressing APCs [32]. These regulatory T cells arise during antigenic stimulation, do not respond to stimulation, and can block the alloreactivity of autologous T cells *in vitro*. Such regulatory T cells can be generated by membrane-bound HLA-G [33] or soluble HLAG5 [34] and have been detected *in vivo* after transplantation [35]. Development of immunosuppressive functions in CD4+/CD25+/FoxP3+ regulatory T cells is influenced by the adenosine-A2A receptor (A2AR) pathway. The A2AR pathway is believed to mediate a negative-feedback immunosuppressive mechanism that limits collateral tissue damage by activated immune cells during responses against pathogens. A2AR also regulates immunosuppressive T-regulatory cells [36]. Activation of A2AR increases intracellular cAMP, which is a potent inhibitor of the NF-κB pathway downstream of immunoreceptors. Thus, A2AR may contribute to the anti-inflammatory effects of A2AR agonists [37]. Adenosine is an important anti-inflammatory molecule. A2AR agonists may protect the kidneys from AKI by inhibiting leukocyte function, which may be further improved by NGAL stimulation.

NGAL is emerging as a mediator of various biological states, such as iron metabolism, innate immunity to bacteria, and kidney development, as well as pathological states such as acute kidney injury, kidney transplant, and chronic kidney disease. The biological role of this molecule remains unclear. Although NGAL serves as a biomarker for many conditions, it appears likely that the extreme sensitivity of detecting NGAL is associated with low specificity for disease conditions, which may hide important biological roles [38]. Such pathways point toward an acute compensatory, protective role for NGAL in response to diverse cellular stresses, including inflammatory and oxidative stress [20], [39], [40], [41]. We have already found some evidence of NGAL involvement in cellular immunity [1].

We have initiated experiments in healthy controls to characterize the possible involvement of NGAL in the immune response and to test the hypothesis that NGAL promotes pro- and anti-inflammatory mechanisms, which may amplify the response of regulatory T cells. In conclusion, the potential role of NGAL as an immunomodulatory molecule has been evaluated and supports a possible role for NGAL in immune tolerance.

In summary, several important results have emerged from this study. (1) NGAL promotes an increase in HLA-G expression in CD4+ T lymphocytes. The role of HLA-G in maternal-fetal tolerance does not end with birth but extends throughout life [42]. NGAL may upregulate HLA-G expression during acute and chronic anti-inflammatory responses [43].(2) Increasing concentrations of NGAL elevate the level of HLA-G expression. NGAL does not increase through a random process. NGAL concentrations increase during various pathological processes and may be modulated, depending on biological need. (3) NGAL does not clearly correlate with iron. HLA-G expression was slightly lower in the absence of iron. The absence of iron slightly reduces NGAL action. The pathological condition of hyposideremia is found during systemic inflammation and anemia and can reduce tissue regeneration and promote aging [44]. (4) NGAL:Ent:Fe proves unable to modify HLA-G expression in the presence of anti-NGAL antibody. Decreased production of NGAL could lower resistance to the phenomena of inflammation and aging. (5) Increasing concentrations of iron-loaded NGAL induce dose-dependent activation of CD4+/CD25+/FoxP3+ cells. Deregulation of T-regulatory cells may cause autoimmune diseases, including multiple sclerosis [45], type 1 diabetes mellitus, myasthenia gravis, systemic lupus erythematosus, autoimmune lymphoproliferative disorders, rheumatoid arthritis, and psoriasis [46], [47]. Thus, NGAL may have an important protective function as an immune activator. T-regulatory cells are important for therapy and tolerance in organ and tissue transplantation [48], [49]. The increased expression of HLA-G that is mediated by NGAL can activate anti-inflammatory and immunological responses by amplifying the T-regulatory cell immune response.

## Supporting Information

Figure S1 **Soluble HLA-G expression.** sHLA-G levels in PHA-activated PBMC cultures were measured by ELISA following treatment with 40-320 ng/ml NGAL:Enterobactin:Iron or 40 ng/ml NGAL:Enterobactin. Values of sHLA-G are expressed in Unit/ml. Means ± SD; n = 3. * p<0.05.
(TIF)

File S1 (DOC)
