# Supplementary figures and images for: Neutrophil Gelatinase-Associated Lipocalin Increases HLA-G+/FoxP3+ T-Regulatory Cell Population in an In Vitro Model of PBMC
Source: PLoS One. 2014 Feb 27;9(2):e89497. doi: 10.1371/journal.pone.0089497 (PMC3937322; doi:10.1371/journal.pone.0089497)

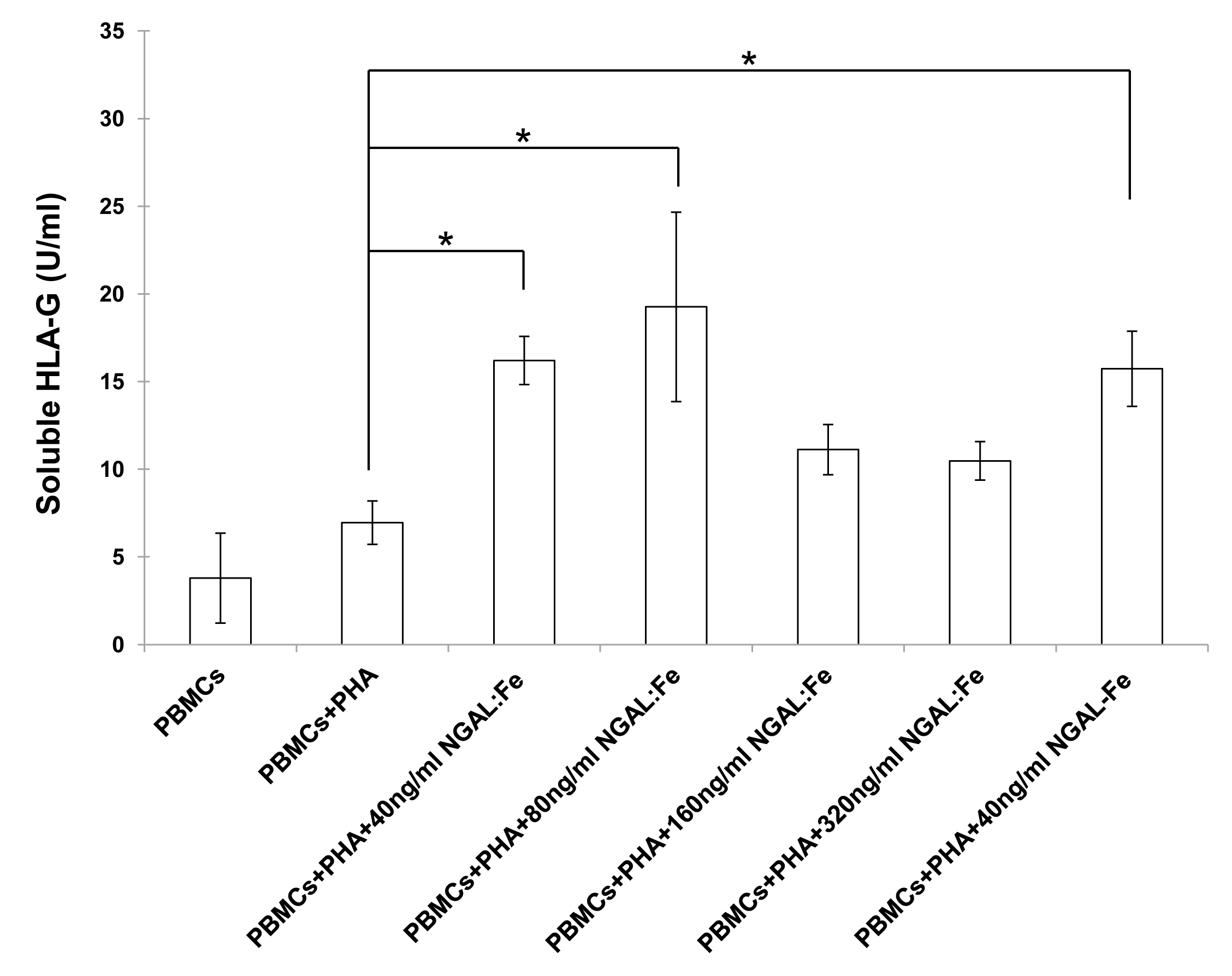

Supplement: Figure S1 — Soluble HLA-G expression. sHLA-G levels in PHA-activated PBMC cultures were measured by ELISA following treatment with 40-320 ng/ml NGAL:Enterobactin:Iron or 40 ng/ml NGAL:Enterobactin. Values of sHLA-G are expressed in Unit/ml. Means ± SD; n = 3. * p<0.05. (TIF) [file pone.0089497.s001.tif]
