# Supplementary material for: Neutrophil Gelatinase-Associated Lipocalin Increases HLA-G+/FoxP3+ T-Regulatory Cell Population in an In Vitro Model of PBMC
Source: PLoS One. 2014 Feb 27;9(2):e89497. doi: 10.1371/journal.pone.0089497 (PMC3937322; doi:10.1371/journal.pone.0089497)
Supplement: File S1 — (DOC) [file pone.0089497.s002.doc]

**Supplemental files**

**NGAL**

NGAL is a 25-kDa protein belonging to the lipocalin family [38]. Lipocalins are a family of small, secreted proteins that act as carriers, primarily transporting small, lipophilic molecules. Several additional functions have been discovered for these proteins, including regulation of cell division, differentiation, cell-to-cell adhesion, and cell survival. NGAL emerges as a mediator of various biological states, such as iron metabolism, innate immunity to bacteria, and kidney development, and pathological states, such as acute kidney injury, kidney transplant, and chronic kidney disease [1].

Evidence of NGAL involvement in iron metabolism has been obtained from crystallographic studies [2]. Further confirmation came from a study by Bao and co-workers, which showed that NGAL binds to iron and catecholamine [44,50]. Iron transport by NGAL induces specific cellular responses. Based on recent studies, a new model of intracellular signaling has been developed, addressing the association of NGAL with its receptors. In certain biological systems, some processes may significantly differ, depending on whether NGAL is associated with iron or not. Various types of endosomal NGAL trafficking have been proposed in relation to cell type and to the association of NGAL with its binding partner [20]. NGAL has an affinity and specificity for ferric bacterial siderophores. Thus, NGAL may be released by neutrophils at the infection or inflammatory site to sequester bacterial ferric siderophores and participate in antibacterial iron depletion, which is activated by the innate immune response [2,3]. In a subsequent study, the ability of NGAL to prevent growth of bacterial strains that are dependent on siderophores for iron was confirmed. Genetically-modified Lcn2^−/−^ mice have a higher susceptibility to contracting *E. coli* infections, resulting in sepsis and death more frequently than in wild-type mice [51]. In addition, *in vitro* studies suggested that NGAL may be involved in kidney development. Local expression of NGAL regulates epithelial morphogenesis [52]. NGAL administration to early progenitor cells of the metanephric mesenchyme induces proliferation, epithelial differentiation, and generation of structures similar to nephrons that express glomerular, proximal, and distal tubule cell surface markers [27]. NGAL may play a significant role in kidney regeneration and repair following damage from ischemia and reperfusion [17,23]. NGAL is a promising biomarker for early diagnosis, predicting disease severity, therapeutic monitoring, and predicting clinical outcomes. NGAL appears to be one of the most promising markers for AKI detection [17,53,54]. As a readily available parameter, urinary NGAL may guide differential diagnosis and initial therapy in allograft recipients with AKI [13]. Various studies have shown that NGAL is involved in kidney transplantation. NGAL can be used to detect complications before the onset of irreversible renal lesions [53,55]. Further investigations have confirmed the role of NGAL as an accurate indicator of delayed graft function in the early post-transplant period [56].

NGAL can be a useful instrument for assessing the severity and progression of chronic kidney disease (CKD) in patients with membranous nephropathy [57] and in non-diabetic patients with CKD stages 2 to 4 [58]. Correlations have been shown between the presence of urinary NGAL in patients with advanced CKD, and the glomerular filtration rate, interstitial fibrosis, and tubular atrophy [59].

**HLA-G**

One important and recent scientific quest is to understand the potential role of the HLA-G complex in immunological tolerance. HLA-G is a non-classical HLA class I molecule with an important role at the fetal-maternal interface, preventing fetus recognition and abortion [60]. The genetic diversity, expression, structure, and function of HLA-G differs from HLA I molecules. HLA-G is characterized by a limited allelic polymorphism and by restricted tissue distribution. The *HLA-G* gene structure is homologous to that of the HLA-I-complex genes. However, in contrast to HLA I, the primary transcript of *HLA-G* generates seven mRNAs that encode membrane isoforms (HLA-G1, G2, G3, G4) and soluble isoforms (HLA-G5, G6, G7) [61]. The membrane-bound isoform, HLA-G1, and the soluble isoform, HLA-G5, are the most highly expressed and best-studied HLA-G proteins. In contrast to classic HLA I, HLA-G does not appear to significantly stimulate the immune system. Responses directed against allogeneic HLA-G have not been reported. However, like HLA class I molecules, HLA-G is able to bind to inhibitory receptors. The following receptors have been described: ILT2/CD85j/LILRB1 (ILT2), ILT4/CD85d/LILRB2 (ILT4), and KIR2DL4/CD158d (KIR2DL4) [62-64]. HLA-G can interact with B cells, T cells, NK cells, and APC through these differentially expressed receptors [42].

HLA-G molecules play key roles in maternal-fetal tolerance [65]. and in preeclampsia, which is characterized by a low level of HLA-G [66-68]. Constitutive HLA-G expression has been found in immune-privileged organs in association with tolerogenic properties via interaction with inhibitory receptors on dendritic, natural killer, and T cells. Immunomodulatory properties of mesenchymal stem cells (MSCs) prove dependent upon multiple factors, including the HLA-G complex. A common inhibitory capacity of MSCs and HLA-G emerges [69]. Constitutive expression of HLA-G and the co-stimulatory molecule, B7-H4, appears to have a functional role in MSCs [70].

HLA-G may be a key factor mediating the immunosuppressive properties of MSCs. The potential role of HLA-G in sustaining a tolerant environment during organ transplantation can not be underestimated. The immunosuppressive properties of HLA-G suggest possible roles in transplantation tolerance [71]. Stem cells that produce HLA-G and/or regulatory molecules may represent a strategy for preventing acute and chronic rejection of allogeneic transplants [72]. Studies monitoring HLA-G expression in patients undergoing renal transplantation have suggested a potential role for HLA-G as an early diagnostic marker of acute renal rejection and a marker of the immunosuppressive status of transplant recipients [73,74]. A deeper understanding of the immunomodulatory potential of HLA-G molecules is needed in order to improve graft acceptance and to reduce the incidence of rejection.

Potential preventive and therapeutic effects of CD4+/CD25+/FOXP3+ T cells have been investigated. Regulatory T cells have gained much interest in the research community and are considered ideal candidates for monitoring operational tolerance in personalized drug-minimizing therapy [48]. A recent study has shown that FoxP3 expression is required for tolerance to transplanted tissues, while continuous nuclear expression is required in order to maintain tolerance[75].

Supplemental references

50 Bao G, Clifton M, Hoette TM, Mori K, Deng SX, et al(2010) Iron traffics in circulation bound to a siderocalin (Ngal)-catechol complex. Nat Chem Biol 6(8): 602-609.

51 Berger T, Togawa A, Duncan GS, Elia AJ, You-Ten A, et al(2006) Lipocalin 2-deficient mice exhibit increased sensitivity to Escherichia coli infection but not to ischemia-reperfusion injury. Proc Natl Acad Sci U S A 103(6): 1834-1839.

52 Gwira JA, Wei F, Ishibe S, Ueland JM, Barasch J et al (2005) Expression of neutrophil gelatinase-associated lipocalin regulates epithelial morphogenesis in vitro. J Biol Chem 280(9): 7875-7882

53 Mishra J, Dent C, Tarabishi R, Mitsnefes MM, Ma Q et al (2005) Neutrophil gelatinase-associated lipocalin (NGAL) as a biomarker for acute renal injury after cardiac surgery. Lancet 365(9466): 1231-1238.

54 Krawczeski CD, Goldstein SL, Woo JG, Wang Y, Piyaphanee N et al (2011) Temporal relationship and predictive value of urinary acute kidney injury biomarkers after pediatric cardiopulmonary bypass. J Am Coll Cardiol 58(22): 2301-2309.

55 Kusaka M, Kuroyanagi Y, Mori T, Nagaoka K, Sasaki H et al (2008) Serum neutrophil gelatinase-associated lipocalin as a predictor of organ recovery from delayed graft function after kidney transplantation from donors after cardiac death. Cell Transplant 17(1-2): 129-134.

56 Bataille A, Abbas S, Semoun O, Bourgeois É, Marie O et al (2011) Plasma neutrophil gelatinase-associated lipocalin in kidney transplantation and early renal function prediction. Transplantation 15:92(9):1024-1030.

57 Bolignano D, Lacquaniti A, Coppolino G, Donato V, Campo S (2009) Neutrophil gelatinase-associated lipocalin (NGAL) and progression of chronic kidney disease. Clin J Am Soc Nephrol Feb:4(2):337-344.

58 Malyszko J, Malyszko JS, Bachorzewska-Gajewska H, Poniatowski B, Dobrzycki S et al (2009) Neutrophil gelatinase-associated lipocalin is a new and sensitive marker of kidney function in chronic kidney disease patients and renal allograft recipients. Transplant Proc 41(1): 158-161.

59 Nickolas TL, Forster CS, Sise ME, Barasch N, Solá-Del Valle D (2012) NGAL (Lcn2) monomer is associated with tubulointerstitial damage in chronic kidney disease. Kidney Int 82(6):718-722

60 Rouas-Freiss N, Marchal RE, Kirszenbaum M, Dausset J, Carosella ED (1997) The alpha1 domain of HLA-G1 and HLA-G2 inhibits cytotoxicity induced by natural killer cells: is HLA-G the public ligand for natural killer cell inhibitory receptors? Proc Natl Acad Sci U S A 94(10):5249-5254.

61 Carosella ED, Moreau P, Le Maoult J, Le Discorde M, Dausset J et al(2003) HLA-G molecules: from maternal-fetal tolerance to tissue acceptance. 81:199-252.

62 Colonna M, Navarro F, Bellón T, Llano M, García P et al (1997) A common inhibitory receptor for major histocompatibility complex class I molecules on human lymphoid and myelomonocytic cells. J Exp Med 186(11):1809-1818.

63 Colonna M, Samaridis J, Cella M, Angman L, Allen RL et al(1998) Human myelomonocytic cells express an inhibitory receptor for classical and nonclassical MHC class I molecules. J Immunol 160(7): 3096-3100.

64 Rajagopalan S, Long EO(2012) KIR2DL4 (CD158d): An activation receptor for HLA-G. Front Immunol 3:258.

65 Rouas-Freiss N, Gonçalves RM, Menier C, Dausset J, Carosella ED (1997) Direct evidence to support the role of HLA-G in protecting the fetus from maternal uterine natural killer cytolysis. Proc Natl Acad Sci U S A 94(21):11520-11525.

66 Hackmon R, Koifman A, Hyodo H, Glickman H, Sheiner E (2007) Reduced third-trimester levels of soluble human leukocyte antigen G protein in severe preeclampsia. Am J Obstet Gynecol 197(3):255.

67 Yie SM, Li LH, Li YM, Librach C (2004) HLA-G protein concentrations in maternal serum and placental tissue are decreased in preeclampsia. Am J Obstet Gynecol 191(2): 525-529.

68. Zhu X, Han T, Yin G, Wang X, Yao Y (2012) Expression of human leukocyte antigen-G during normal placentation and in preeclamptic pregnancies. Hypertens Pregnancy 31(2):252-260.

69 Lila N, Rouas-Freiss N, Dausset J, Carpentier A, Carosella ED et al (2001). Soluble HLA-G protein secreted by allo-specific CD4+ T cells suppresses the allo-proliferative response: a CD4+ T cell regulatory mechanism. Proc Natl Acad Sci U S A 98(21):12150-12155.

70 Gebler A, Zabel O, Seliger B (2012) The immunomodulatory capacity of mesenchymal stem cells. Trends Mol Med 18(2):128-134.

71 Wu J, Zhang W, Hernandez-Lopez P, Fabelo E, Parikh M et al(2009) Isoforms of human leukocyte antigen-G and their inhibitory receptors in human kidney allograft acceptance. Hum Immunol 70(12):988-994.

72 Deschaseaux F, Delgado D, Pistoia V, Giuliani M, Morandi F et al (2010) HLA-G in organ transplantation: towards clinical applications. Cell Mol Life Sci 68(3): 397-404.

73 Lu N, Zhang Y, Zou X, Yang X, Tian J et al (2012) HLA-G on peripheral blood CD4(+) T lymphocytes: a potential predictor for acute renal rejection. Transpl Int 24(11):1103-11011.

74 Qiu J, Terasaki PI, Miller J, Mizutani K, Cai J et al (2006) Soluble HLA-G expression and renal graft acceptance. Am J Transplant 6(9):2152-2156.

75 Regateiro FS, Chen Y, Kendal AR, Hilbrands R, Adams E et al (2012) Foxp3 expression is required for the induction of therapeutic tissue tolerance. J Immunol 189(8):3947-3956.
